# Multiple factors co-limit short-term in situ soil carbon dioxide emissions

**DOI:** 10.1371/journal.pone.0279839

**Published:** 2023-02-15

**Authors:** James W. Raich, Mark S. Kaiser, Mathew E. Dornbush, Jonathan G. Martin, O. J. Valverde-Barrantes

**Affiliations:** 1 Department of Ecology, Evolution and Organismal Biology, Iowa State University, Ames, Iowa, United States of America; 2 Department of Statistics, Iowa State University, Ames, Iowa, United States of America; 3 Cofrin School of Business, University of Wisconsin-Green Bay, Green Bay, Wisconsin, United States of America; 4 Natural Resources, Northland College, Ashland, Wisconsin, United States of America; 5 Institute of Environment, Department of Biological Sciences, Florida International University, Miami, Florida, United States of America; Jinan University, CHINA

## Abstract

Soil respiration is a major source of atmospheric CO_2_. If it increases with warming, it will counteract efforts to minimize climate change. To improve understanding of environmental controls over soil CO_2_ emission, we applied generalized linear modeling to a large dataset of in situ measurements of short-term soil respiration rate, with associated environmental attributes, which was gathered over multiple years from four locations that varied in climate, soil type, and vegetation. Soil respiration includes many CO_2_-producing processes: we theorized that different environmental factors could limit each process distinctly, thereby diminishing overall CO_2_ emissions. A baseline model that included soil temperature, soil volumetric water content, and their interaction was effective in estimating soil respiration at all four locations (*p* < 0.0001). Model fits, based on model log likelihoods, improved continuously as additional covariates were added, including mean daily air temperature, enhanced vegetation index (EVI), and quadratic terms for soil temperature and water content, and their interactions. The addition of land cover and its direct interactions with environmental variables further improved model fits. Significant interactions between covariates were observed at each location and at every stage of analysis, but the interaction terms varied among sites and models, and did not consistently maintain importance in more complex models. A main-effects model was therefore tested, which included soil temperature and water content, their quadratic effects, EVI, and air temperature, but no interactions. In that case all six covariates were significant (*p* < 0.0001) when applied across sites. We infer that local-scale soil-CO_2_ emissions are commonly co-limited by EVI and air temperature, in addition to soil temperature and water content. Importantly, the quadratic soil temperature and moisture terms were significantly negative: estimated soil-CO_2_ emissions declined when soil temperature exceeded 22.5°C, and as soil moisture differed from the optimum of 0.27 m^3^ m^-3^.

## Introduction

Soils across the globe produce CO_2_ via respiration by the organisms they contain. The resulting CO_2_ emissions, soil respiration (*Rsoil*), comprise the second largest land–to–atmosphere CO_2_ flux, after total ecosystem respiration. Soil respiration varies temporally and spatially, often in parallel with abiotic factors such as temperature and water availability. Plants contribute substantial carbon to soils and, like *Rsoil*, are influenced by temperature and soil water content. The concomitant uptake, production, and release of CO_2_ from plants and soils confounds the interpretation of atmospheric CO_2_ measurements. For example, seasonally maximum soil emissions coincide with declining atmospheric CO_2_ concentrations during northern-hemisphere summers, when photosynthetic CO_2_ assimilation exceeds respiratory CO_2_ production [1], but the atmosphere records only their net effect. Changes in climate, land cover, plant growth, and their interactions are all likely to modify soil-CO_2_ emissions in the future, but the magnitude and timing of potential changes remain uncertain [2–4]. Given that soils release about 85 Pg of CO_2_-C into the atmosphere each year (range = 68–94 Pg y^-1^) [5–10], even relatively small changes in *Rsoil* can meaningfully alter the atmosphere’s warming potential.

Properly characterizing environmental influences on soil respiration is challenging, however, because *Rsoil* is a compound flux that derives from multiple organisms and processes [11]. Soils, for instance, produce CO_2_ via the decay of detritus within the litter layer (O horizon), the soil matrix, and dead wood, each of which forms a distinct environment. Carbon dioxide is produced by soil microbes, protozoans, fungi, worms, and arthropods; via the respiration of roots and mycorrhizae; and, in some cases, from abiotic weathering of carbonates. Each of those organisms and processes may respond to the environment in distinct ways. For instance, roots and soil organic matter respond differently to soil temperature [12]; different soil heterotrophs differ in their responses to low soil water potentials [13]; and soil microbial activity persists in soils that are drier than the plant wilting point [14, 15].

Photosynthetic CO_2_ assimilation directly sustains *Rsoil* by providing the carbon that fuels it. Global terrestrial net primary productivity averages ~56 Pg of C annually (range 46–65) [16–26]. That indicates that about 65% of global soil-CO_2_ emissions derive from the decomposition of above- and belowground detritus, and about 35% derive from the respiration of roots and mycorrhizae, or soil autotrophic respiration. Therefore, factors that influence plant photosynthesis, carbon transport to roots, root turnover, and root-rhizosphere respiration also affect *Rsoil*. Greater canopy photosynthesis, if accompanied by carbon sequestration in biomass or soils, provides a potentially important brake on increasing atmospheric CO_2_ levels [27–30]. Over 2009–2018, net C accumulation by global vegetation averaged an estimated 3.2 Pg y^-1^ of C, which more than offset net land use-change losses of 1.5 Pg y^-1^ [31], but whether C sequestration can continue to increase in parallel with warming-enhanced soil-CO_2_ emission rates is unclear [32, 33], especially with increasingly pervasive human land use [e.g., 34]. The complexity of soil carbon-cycling processes makes robust predictions of soil responses to environmental changes difficult.

Multiple environmental factors influence plant photosynthesis, the partitioning of photosynthates to above- and belowground processes, detritus production, and soil carbon cycling. Environmental change is similarly multifactorial, as it includes climatic and atmospheric changes, changes in the distribution and abundance of biota, and alterations in land cover and land use. Determining how environmental changes will influence terrestrial carbon cycling is a complex challenge. Soil respiration is central to that challenge, as a result of its magnitude, and because it is sensitive to environmental conditions. Importantly, soil respiration is measured directly, in contrast to canopy photosynthesis and ecosystem respiration. Soil respiration measurements across environmental gradients provide a valuable resource for model testing and improvement. We theorize that the multiple individual processes and organisms that contribute to *Rsoil* generate the potential for limitation of any single contributing process, by any environmental condition, to limit total CO_2_ emissions from soils. That is, in a manner analogous to multiple potential nutrient limitations to plant growth [35], we posit that environmental conditions that diminish soil-CO_2_ production by any important contributor to soil respiration, will lower total soil respiration.

To test this idea, we applied sequentially more complex mathematical models for estimating observed in situ soil CO_2_ emission rates, based on paired measurements of fluxes and environmental attributes, to identify statistically important covariates of *Rsoil* within and across sites, and to characterize the forms of their relationships to fluxes. Individual sites experience limited ranges of environmental variability. To broaden the robustness and usefulness of our findings, we gathered a multisite dataset that included only fully comparable *Rsoil* measurements. We were specifically interested in determining (Objective 1) whether *Rsoil* continued to increase at ever-warmer temperatures, when the joint influences of other environmental covariates were included. Completing that objective required that we (Objective 2) identify the cofactors that meaningfully improved fits of models that already included temperature, across ecosystem types and through time, and thus might influence the observed *Rsoil* × temperature relationship. Those objectives allowed us to (Objective 3) identify a common model form that included only consistently influential covariates, even if that was not the model of choice for any single location. We focused on identifying the covariates that consistently improved overall model fits, and the forms of those fits, rather than defining parameter values, which are specific to input datasets.

## Methods

### Study sites

We utilized data from four study sites that encompassed a range of ecosystem types, climatic conditions, and CO_2_ efflux rates (Table 1): La Selva, Costa Rica; Rhodes Farm, Iowa, USA; the Chequamegon National Forest in northern Wisconsin, USA; and Bear Creek, Iowa, USA. Replicated-block field experiments were conducted at the first two locations; observational comparisons were made at the other two. At each location, the primary experimental or observational treatment was land cover, as characterized by the plant community present: soil characteristics, climate and remotely sensed parameters varied among locations. Our research in Costa Rica was undertaken in full collaboration with the La Selva Biological Station of the Organization for Tropical Studies (https://tropicalstudies.org), which owns and manages the site and was collaboratively funded in support of this research. No biological samples or protected species were collected or exported. The research was approved and permitted by the Costa Rican Sistema Nacional de Áreas de Conservación, Ministerio del Ambiente y Energía, Pasaporte Científico number 084544671.

**Table 1:** Environmental characteristics of the four study sites.

Location	Bear Creek	Chequamegon	La Selva	Rhodes Farm
Latitude, Longitude	42.19°N, 93.49°W	45.8ºN, 90.07ºW	10.43ºN, 84.04ºW	41.89ºN, 93.205ºW
Elevation (m)	319	520	70	317
Climate^a^	Dfa	Dfb	Af	Dfa
MODIS land cover^b^	12	4, 5	2	12
MAT (°C)^c^	8.6	4.0	25.1	8.7
MAP (mm)^d^	910	787	4540	876
Soil Order	Mollisol	Spodosol	Ultisol	Entisol
Dates	2001–2002	2000–2004	2004–2008	2003–2006
Vegetation	Grasslands	Temperate Forests	Tree Plantations	Grasslands
Soil temperature range (°C)	-0.1–36.2	1.8–24.0	21.9–28.6	-1.6–33.1
Soil moisture range (m^3^ m^-3^)	0.04–0.66	0.03–0.87	0.07–0.77	0.12–0.45
EVI range^e^	0.10–0.67	0.18–0.72	0.44–0.63	0.18–0.63
Air temperature range (°C)	-1.7–29.1	4.7–27.5	21.5–31.0	-10.3–28.6

The specified ranges for soil temperature, soil moisture, EVI and air temperature refer only to dates with *Rsoil* measurements.

^a^ Köppen-Geiger classification [36]: Af is a fully humid equatorial climate; Dfa is a seasonal temperate climate with snowy winters and hot summers; and Dfb is a seasonal temperate climate with snowy winters and warm summers.

^b^ The locally dominant land cover based on remotely sensed Moderate Resolution Imaging Spectroradiometer data (https://modis.gsfc.nasa.gov/about/): 2 = Evergreen Broadleaf Forest, 4 = Deciduous Broadleaf Forests, 5 = Mixed Forests, 12 = Croplands.

^c^ Mean annual air temperature.

^d^ Mean annual precipitation.

^e^ Remotely sensed enhanced vegetation index [37].

At Rhodes Farm we conducted a randomized-block field experiment that compared the effects of C_3_ versus C_4_ grass species on ecosystem-level carbon-cycling processes [38–40]. The original vegetation there was broad-leaved deciduous riparian forest that was converted to cropland early in the 20^th^ century. The experimental site was cultivated approximately annually for at least 70 years before the experiment was established, usually for maize that was harvested for cattle fodder. The soils were Mollic Udifluvents derived from loess. The experiment contained four 15 m × 90 m blocks that paralleled Clear Creek. Blocks were bisected into C_3_ and C_4_ treatments. Within each treatment, three different species of perennial grass species were planted in monoculture in 15 m × 15 m plots. One of the C_4_ species did not establish, leaving each block with three C_3_ and two C_4_ grass species.

We also analyzed measurements from a randomized-block experiment that was established in 1988–1989 on newly abandoned pastures at the La Selva Biological Station in the Atlantic lowlands of Costa Rica. The experiment is described by [41, 42]. The soils were Typic Tropohumults in the Matabuey consociation [43]. The native vegetation was species-rich rainforest [44] that had been cleared and converted to pasture in the 1950s. We utilized data from plots of four planted, native, broad-leaved tree species that retained relatively full canopies in all four blocks: *Hieronyma alchorneoides* Allemão, *Pentaclethra macroloba* (Willd.) Kunth., *Virola koschnyi* Warb., and *Vochysia guatemalensis* Donn.Sm. The plantations were 16 years old at the beginning of this study, and each plot measured 50 m × 50 m. Seedlings were planted at a 3-m × 3-m spacing, with 256 seedlings per plot. The data used herein are a subset of a larger online dataset [45, 46].

The Chequamegon National Forest is dominated by northern hardwood forests but includes a variety of forest types and ages, which were revisited through time to characterize seasonal dynamics [47, 48]. The dataset we used included: temperate coniferous forests dominated by hemlock (*Tsuga canadensis* (L.) Carrière or red pine (*Pinus resinosa* Aiton); temperate deciduous forests dominated by deciduous broad-leaved tree species; mixed deciduous-coniferous forests; *Populus tremuloides* Michx. (aspen) stands of different ages; and recently clearcut sites. The study sites were within the airshed of the Willow Creek flux tower [49, 50] and were interspersed with wetlands and lakes. The soils were sandy loams on glacial till plains and moraines left from the Wisconsin glaciation.

The Bear Creek study was in Story County, Iowa, on several farms that participated in riparian-zone management activities [51]. The experiment was observational with replicated plots in each of multiple land-cover types [52, 53]. Most of the plots utilized in this current study were on poorly drained, fine-loamy, mesic Cumulic Endoaquolls. We measured *Rsoil* in three plots in each of five anthropogenic grassland types that were <1 to >50 years in age and were dominated by either C_3_ or C_4_ grasses, defining two land-cover treatments.

### Soil fluxes and ancillary measurements

We measured soil-CO_2_ emission rates with LI-COR soil efflux measurement systems (LI-COR, Lincoln, Nebraska, USA) in parallel with concomitant and co-located measurements of surface-soil temperature and surface-soil volumetric water content (VWC). We did not include measurements from frozen soils or from snow-covered sites, which represent physically different conditions. Similar protocols were applied in the different studies. The fundamental measurement—one sample—consisted of a CO_2_ flux measurement from one chamber at one time in one plot, with concomitant measurements of surface-soil temperature and surface-soil volumetric water content. Each flux estimate was based on two-three sequential measurements that were averaged, following the recommendations of the manufacturer. All measurements were gathered between 06:00 and 18:00. Instruments were carried throughout the study sites to access the different blocks, treatments, and plots, which were sometimes distant from one another. The dataset analyzed for this study is openly available [54].

Field measurements of soil CO_2_ fluxes at La Selva are described in [41, 42, 45, 55]. Soil respiration was initially measured with an LI-6400 System attached to a 6400–09 Soil CO_2_ Flux Chamber that was placed onto 5-cm tall, 10-cm diameter soil collars. Later measurements were collected with an LI-8100A Automated Soil CO_2_ Flux System attached to an 8100–103 chamber, with 20-cm diameter soil collars. Soil temperature was measured with a soil temperature probe that was pushed 5–10 cm into the soil next to each collar at the time of flux measurement. Soil moisture was measured at the same time to 12-cm depth with time domain reflectometry (CS-615 Soil Moisture Probe, Campbell Scientific, Logan, Utah, USA). The entire collection of soil respiration data from that site is available online [46]: we herein used data collected in four plantation types over 45 across-site surveys and totaling 2497 individual flux measurements.

At Rhodes Farm, soil respiration was measured with an LI-6400 System attached to a 6400–09 flux chamber that was placed on 10-cm diameter soil collars that were randomly pre-positioned in each plot of each block. The study encompassed five plots in each of four blocks, and one or two soil collars were placed in each plot prior to flux measurements. Soil temperature was measured beside each chamber at the time of flux measurement. Soil moisture was monitored in the 0–15 cm surface soil horizon with MoisturePoint soil-water profiling probes (E.S.I. Environmental Sensors Inc., www.esica.com). We initially installed one probe in the center of each block, and later increased that to one probe per plot. We herein used data from 48 across-site measurement surveys, which included 1543 flux determinations.

Soil carbon dioxide fluxes in different forest types in the Chequamegon National Forest are described in [47, 48]. Soil respiration was measured with LI-COR 6400–09 Soil CO_2_ Flux Chamber placed on 5-cm tall, 10-cm diameter soil collars that were placed at eight new, random locations per plot for each of the measurement periods. An integrated temperature probe (steel-embedded copper-Constantan thermocouple, type T) was used to record soil temperature down to 10 cm by each soil respiration collar. Volumetric soil moisture down to 15 cm was determined by each collar by time domain reflectometry using a portable soil moisture probe (CS615 Water Content Reflectometer, Campbell Scientific, Logan, Utah, USA). We herein used data from 67 surveys that included 2667 individual measurements of soil-CO_2_ efflux.

At Bear Creek, Iowa, we used and LI-8100A Automated Soil CO_2_ Flux System attached to an 8100–102 automated flux chamber, which fit over 10-cm diameter soil collars. Soil temperature was measured next to each chamber at the time of flux measurement with a thermocouple probe. At Bear Creek, soil gravimetric (GWC, g water per g soil) rather than volumetric water content was measured. Gravimetric data were transformed to volumetric water contents (VWC, m m^-1^) based on soil bulk densities (BD, Mg m^-3^) that were measured using volumetric samplers with four samples per plot and corrected to standard units:

soilVWC=soilGWC×SoilBD (1)

The treatments at Bear Creek represented riparian buffer strips that were planted along Bear Creek on multiple active farms, and they had different ages and histories. We distinguished mixed C_3_ grasslands, which contained a mixture of cool-season grasses, from plantings of C_4_ prairie grasses, which included some prairie forbs. We utilized a total of 1332 flux measurement from three different plots in each of five different ages of grasslands for this study.

Our final cumulative *Rsoil* dataset included 8039 chamber-level determinations of soil-CO_2_ emission rates, each with paired measurements of: date, soil temperature (*Tsoil*), soil VWC, the location of the site, and the experimental or observational treatment (land cover). To that dataset we appended measurements of air temperature (*Tair*) and enhanced vegetation index (EVI). Mean daily air temperature data was downloaded from weather stations located near each site; daily minimum and maximum temperature were averaged to estimate the daily mean when necessary. To characterize the seasonal dynamics of canopy development we downloaded remotely sensed EVI from NASA’s MOD13Q1 product (Band 250m_16_days_EVI, https://daac.ornl.gov/) for the time periods covering soil flux measurements at each site [56]. Imagery for Rhodes Farm was centered on 41.890° N, 93.205° W and covered 2.25 × 2.25 km. Imagery for La Selva were centered at 10.4299° N, 84.0363° W, covered 2.25 x 2.25 km. Data for the Chequamegon National Forest were centered at 45.8º N, 90.0667º W and encompassed 6.25 x 6.25 km. Data for Bear Creek were centered over 42.191° N, 93.4897° W and covered 2.25 x 2.25 km. We used linear interpolation between imaging dates to estimate the EVI on dates when *Rsoil* was measured. In all cases multiple land cover types were included within the imaged areas, so EVI data refer to study-site locations, whereas vegetation- and soil-attribute data pertain to within-location plots.

### Statistical analyses

We examined marginal pairwise associations among *Rsoil* and *Tsoil*, VWC, *Tair*, and EVI using traditional Pearson product-moment correlations (*r*). We used linear regression as the basic tool of statistical analysis, implemented using JMP (2019, SAS Institute Inc., Cary, NC, USA. www.jmp.com). Statistical analyses were based on the response variable *log*(*Rsoil)*, with *Rsoil* expressed as micromol cm^-2^ s^-1^ of C, because variability in emission rates increased with temperature. We fit a sequence of increasingly complex models using *log*(*Rsoil*) as the response variable. Each step consisted of testing a null hypothesis that was evaluated four times, once at each location, in fulfillment of our objectives: “The addition of *variable x* does not improve the fit of the model.” At a given location (Rhodes Farm, La Selva, Chequamegon, or Bear Creek), let *Y*_*i*_ denote log(*Rsoil*) for observation *i* = 1, …, *n*. Let *x*_1,*i*_ denote *Tsoil* for this same observation, let *x*_2,*i*_ denote VWC, and let *x*_3,*i*_ denote EVI. Let ecosystem types at a given location be indexed by *v* = 1, …, *V*. Define a set of indicator variables as, for *v* = 1, …, *V*– 1:

$$z_{v,i}=\left\{\begin{array}{ll} 1 & if\;Ecosystem\;type\;for\;observation\;i\;was\;v\\ 0 & otherwise \end{array}\right.$$

The four basic models used for each location follow. Our baseline model included covariates of soil temperature and soil water content:

Model *i*
$$Y_{i}=\beta_{0}+\beta_{1}\;\chi_{1,i}+\beta_{2}\;\chi_{2,i}+\lambda_{12}\;\chi_{1,i}\;\chi_{2,i}+\in_{\text{i}}$$

In model (*i*) and all that follow, the error terms (*ϵ*_*i*_) are assumed to be independent and identically distributed with *N*(0, sigma^2^) distributions. To test for possible effects of canopy photosynthesis and carbon supply to roots, we next incorporated EVI and *Tair* and compared model *i* to:

Model *ii*
$$Y_{i}=\beta_{0}+\beta_{1}\;\chi_{1,i}+\beta_{2}\;\chi_{2,i}+\beta_{3}\;\chi_{3,1}+\beta_{4}\;\chi_{4,1}+\lambda_{1,2}\;\chi_{1,i}\;\chi_{2,i}+\gamma_{1,3}\;\chi_{1,i}\;\chi_{3,i}+\gamma_{1,4}\;\chi_{1,i}\;\chi_{4,i}+\gamma_{2,3}\;\chi_{2,i}\;\chi_{3,i}+\gamma_{2,4}\;\chi_{2,i}\;\chi_{4,i}+\gamma_{3,4}\;\chi_{3,i}\;\chi_{4,i}+\gamma_{1,2,3}\;\chi_{1,i}\;\chi_{2,i}\;\chi_{3,i}+\gamma_{1,2,4}\;\chi_{1,i}\;\chi_{2,i}\;\chi_{4,i}+\gamma_{1,3,4}\;\chi_{1,i}\;\chi_{3,i}\;\chi_{4,i}+\gamma_{2,3,4}\;\chi_{2,i}\;\chi_{3,i}\;\chi_{4,i}+\gamma_{1,2,3,4}\;\chi_{1,i}\;\chi_{2,i}\;{\chi_{3,i}\;\chi }_{4,i}+\in_{i}$$

To test for non-linearity in the soil temperature and soil moisture relationships, we added quadratic terms for each:

Model *iii*
$$Y_{i}=\beta_{0}+\beta_{1}\;\chi_{1,i}+\beta_{2}\;\chi_{2,i}+\beta_{3}\;\chi_{3,i}+\beta_{4}\;\chi_{4,i}+\alpha_{1}\;\chi_{1,i}^{2}+\alpha_{2}\;\chi_{2,i}^{2}+\lambda_{1,2}\;\chi_{1,i}\;\chi_{2,i}+\lambda_{1,3}\;\chi_{1,i}\;\chi_{3,i}+\lambda_{1,4}\;\chi_{1,i}\;\chi_{4,i}+\lambda_{2,3}\;\chi_{2,i}\;\chi_{3,i}+\lambda_{2,4}\;\chi_{2,i}\;\chi_{4,i}+\lambda_{3,4}\;\chi_{3,i}\;\chi_{4,i}+\lambda_{1,2,3}\;\chi_{1,i}\;\chi_{2,i}\;\chi_{3,i}+\lambda_{1,2,4}\;\chi_{1,i}\;\chi_{2,i}\;\chi_{4,i}+\lambda_{1,3,4}\;\chi_{1,i}\;\chi_{3,i}\;\chi_{4,i}+\lambda_{2,3,4}\;\chi_{2,i}\;\chi_{3,i}\;\chi_{4,i}+\lambda_{1,2,3,4}\;\chi_{1,i}\;\chi_{2,i}\;\chi_{3,i}\;\chi_{4,i}+\in_{i}$$

Finally, to determine whether land cover influenced observed fluxes, we considered:

Model *iv*
$$Y_{i}=\beta_{0}+\beta_{1}\;\chi_{1,i}+\beta_{2}\;\chi_{2,i}+\beta_{3}\;\chi_{3,i}+\beta_{4}\;\chi_{4,i}+\alpha_{1}\;\chi_{1,i}^{2}+\alpha_{2\;}\chi_{2,i}^{2}+\lambda_{1,2}\;\chi_{1,i}\;\chi_{2,i}+\lambda_{1,3}\;\chi_{1,i}\;\chi_{3,i}+\lambda_{1,4}\;\chi_{1,i}\;\chi_{4,i}+\lambda_{2,3}\;\chi_{2,i}\;\chi_{3,i}+\lambda_{2,4}\;\chi_{2,i}\;\chi_{4,i}+\lambda_{3,4}\;\chi_{3,i}\;\chi_{4,i}+\lambda_{1,2,3}\;\chi_{1,i\;}\;\chi_{2,i}\;\chi_{3,i}+\lambda_{1,2,4}\;\chi_{1,i}\;\chi_{2,i}\;\chi_{4,i}+\lambda_{1,3,4}\;\chi_{1,i}\;\chi_{3,i}\;\chi_{4,i}+\lambda_{2,3,4}\;\chi_{2,i}\;\chi_{3,i}\;\chi_{4,i}+\lambda_{1,2,3,4}\;\chi_{1,i}\;\chi_{2,i}\;\chi_{3,i}\;\chi_{4,i}+\left(\sum_{v=1}^{v-1}\emptyset_{v}\;z_{v,i}\right)+\left(\sum_{v=1}^{v-1}\gamma_{1,v}\;\chi_{1,i}\;z_{v,i}\right)+\left(\sum_{v=1}^{v-1}\gamma_{2,v}\;\chi_{2,i}\;z_{v,i}\right)+\left(\sum_{v=1}^{v-1}\gamma_{3,v}\;\chi_{3,i}\;z_{v,i}\right)+\left(\sum_{v=1}^{v-1}\gamma_{4,v}\;\chi_{4,i}\;z_{v,i}\right)+\in_{i}$$

Note that in model *iv* we have grouped terms involving land cover as main effect terms in the first parentheses, interactions between land cover and soil temperature in the second parentheses, interactions between land cover and soil water content in the third parentheses, interactions between land cover and EVI in the fourth parentheses, and interactions between land cover and air temperature in the fifth parentheses. Likelihood ratio tests for the significance of effects in the results that follow were conducted in terms of these groups of parameters.

Our strategy was to begin with a basic model formulated around the major factors of soil temperature and soil moisture, and then examine the effect of adding additional covariates. Note that we are primarily interested in which terms should be deemed meaningful rather than the estimated regression coefficients of those terms. Particularly in models that contain polynomial terms, interpretation of regression coefficients can be difficult if not impossible. The models given in (*i*-*iv*) were fit to data from individual locations. They were also fit to a combined data set containing all the observations from all four locations. We were especially interested in any differences found in the sets of meaningful model terms between the combined and location-specific (individual) data sets.

Estimation and testing of regression models were conducted within the framework of generalized linear models (*glm*) using normal random components and identity link functions. Formal tests between models were conducted as likelihood ratio tests, and tests of individual model parameter significance were conducted as Wald tests, the asymptotic bases of these procedures being justified by the large sample sizes at each of our four individual locations (*N* = 1332–2667). The Akaike information criterion (AIC) was also used in model comparison. Plots of deviance residuals against fitted values were examined as a diagnostic for model performance.

## Results

### Correlation analyses

Soil-CO_2_ emission rates correlated significantly and positively with *Tsoil*, *Tair*, and EVI at all four locations, but negatively with VWC (Table 2). Significantly positive correlations were also observed between *Tsoil* and EVI, *Tsoil* and *Tair*, and between EVI and *Tair*. Negative correlations were consistently observed between *Tsoil* and VWC, VWC and *EVI*, and between VWC and *Tair* (Table 2). Given the patterns of correlation observed among the variables that were potential covariates, we expected regressions with multiple covariates to display collinearity of effects, which contributes to our focus on which terms appear meaningful in models, rather than estimation of the numerical coefficients associated with those terms.

**Table 2:** Pearson product-moment correlations (*r*) between individual measurements of *Rsoil* and paired measurements of environmental variables at each of the four study sites.

	Bear Creek	Chequamegon	La Selva	Rhodes Farm
Sample size (*N*)	1332	2667	2497	1543
*Log*(*Rsoil*) × *Tsoil*	0.82 (<0.0001)	0.75 (<0.0001)	0.10 (<0.0001)	0.77 (<0.0001)
*Log*(*Rsoil*) × VWC	-0.34 (<0.0001)	-0.04 (0.02)	-0.25 (<0.0001)	-0.16 (<0.0001)
*Log*(*Rsoil*) × EVI	0.70 (<0.0001)	0.61 (<0.0001)	0.13 (<0.0001)	0.74 (<0.0001)
*Log*(*Rsoil*) × *Tair*	0.83 (<0.0001)	0.66 (<0.0001)	0.13 (<0.0001)	0.79 (<0.0001)
*Tsoil* × VWC	-0.50 (<0.0001)	-0.15 (<0.0001)	-0.13 (<0.0001)	-0.16 (<0.0001)
*Tsoil* × EVI	0.82 (<0.0001)	0.71 (<0.0001)	0.18 (<0.0001)	0.80 (<0.0001)
*Tsoil* × *Tair*	0.93 (<0.0001)	0.82 (<0.0001)	0.67 (<0.0001)	0.89 (<0.0001)
VWC× EVI	-0.54 (<0.0001)	-0.09 (0.0003)	-0.32 (<0.0001)	-0.54 (<0.0001)
VWC × *Tair*	-0.12 (<0.0001)	-0.19 (<0.0001)	-0.11 (<0.0001)	-0.42 (<0.0001)
EVI × *Tair*	0.73 (<0.0001)	0.14 (<0.0001)	0.74 (<0.0001)	0.78 (<0.0001)

Units for *Rsoil* were as measured, in μmol cm^-2^ s^-1^. Environmental covariates included surface-soil temperature (*Tsoil*, °C), surface-soil volumetric water content (VWC, m^3^ m^-3^), enhanced vegetation index (EVI) and mean daily air temperature (*Tair*, °C). Correlation probabilities are in parentheses.

### Regression analyses

Models *i*-*iv* were fit to data from each location individually, and a summary of the results is contained in Table 3, with parameter *p* values shown in S1 Table. Throughout, we refer to comparisons with an associated significance level of *p* < 0.05 as “significant”, although actual *p* values will be reported wherever possible. Residual plots are commented on, largely without discussion.

**Table 3:** Summary results of models fitted to data from four locations.

Model	Bear Creek	Chequamegon	La Selva	Rhodes Farm	Combined^a^
	*N* = 1332	*N* = 2667	*N* = 2497	*N* = 1543	*N* = 8039
Model *i*; variables *Tsoil*, VWC					
Parameters	4	4	4	4	4
AIC	2160	2788	2021	1802	10927
Log Likelihood	-1075	-1389	-1006	-896	-5459
model *p*	<0.0001	<0.0001	<0.0001	<0.0001	<0.0001
Model *i* + EVI					
Parameters	8	8	8	8	8
AIC	1940	2709	1972	1474	9217
Log Likelihood	-961	-1345	-977	-728	-4599
model *p*	<0.0001	<0.0001	<0.0001	<0.0001	<0.0001
Model *i* + *Tair*					
Parameters	8	8	8	8	8
AIC	1968	2710	2009	1442	9093
Log Likelihood	-975	-1346	-996	-712	-4537
model *p*	<0.0001	<0.0001	<0.0001	<0.0001	<0.0001
Model *ii*, variables *Tsoil*, VWC, EVI, *Tair*.					
Parameters	16	16	16	16	16
AIC	1831	2671	1937	1263	8637
Log Likelihood	-898	-1318	-951	-614	-4302
model *p*	<0.0001	<0.0001	<0.0001	<0.0001	<0.0001
Model *ii* + *Tsoil* ^2^					
Parameters	17	17	17	17	17
AIC	1820	2628	1928	1258	8409
Log Likelihood	-892	-1296	-946	-611	-4187
model *p*	<0.0001	<0.0001	<0.0001	<0.0001	<0.0001
Model *ii* + VWC ^2^					
Parameters	17	17	17	17	17
AIC	1826	2446	1867	1256	8341
Log Likelihood	-895	-1205	-916	-610	-4153
model *p*	<0.0001	<0.0001	<0.0001	<0.0001	<0.0001
Model *iii*, variables: *Tsoil*, *Tsoil* ^ *2* ^ , VWC, VWC ^ *2* ^ , EVI, *Tair*					
Parameters	18	18	18	18	18
AIC	1817	2400	1857	1251	8170
Log Likelihood	-889	-1181	-909	-606	-4066
model *p*	<0.0001	<0.0001	<0.0001	<0.0001	<0.0001
Model *iv*, variables *Tsoil*, *Tsoil* ^ *2* ^ , VWC, VWC ^ *2* ^ , EVI, *Tair*, CoverType					
Parameters	23	38	33	23	68
AIC	1624	2122	1534	1246	7148
Log Likelihood	-787	-1021	-732	-599	-3505
model *p*	<0.0001	<0.0001	<0.0001	<0.0001	<0.0001

Parameter values of models *i*, *ii*, *iii* and *iv* are presented in S1 Table.

Direct comparisons of increasingly complex models are presented in S2 Table.

^a^ The Combined column, at right, was derived from the data from all four locations without reference to location.

**Fig 1:**
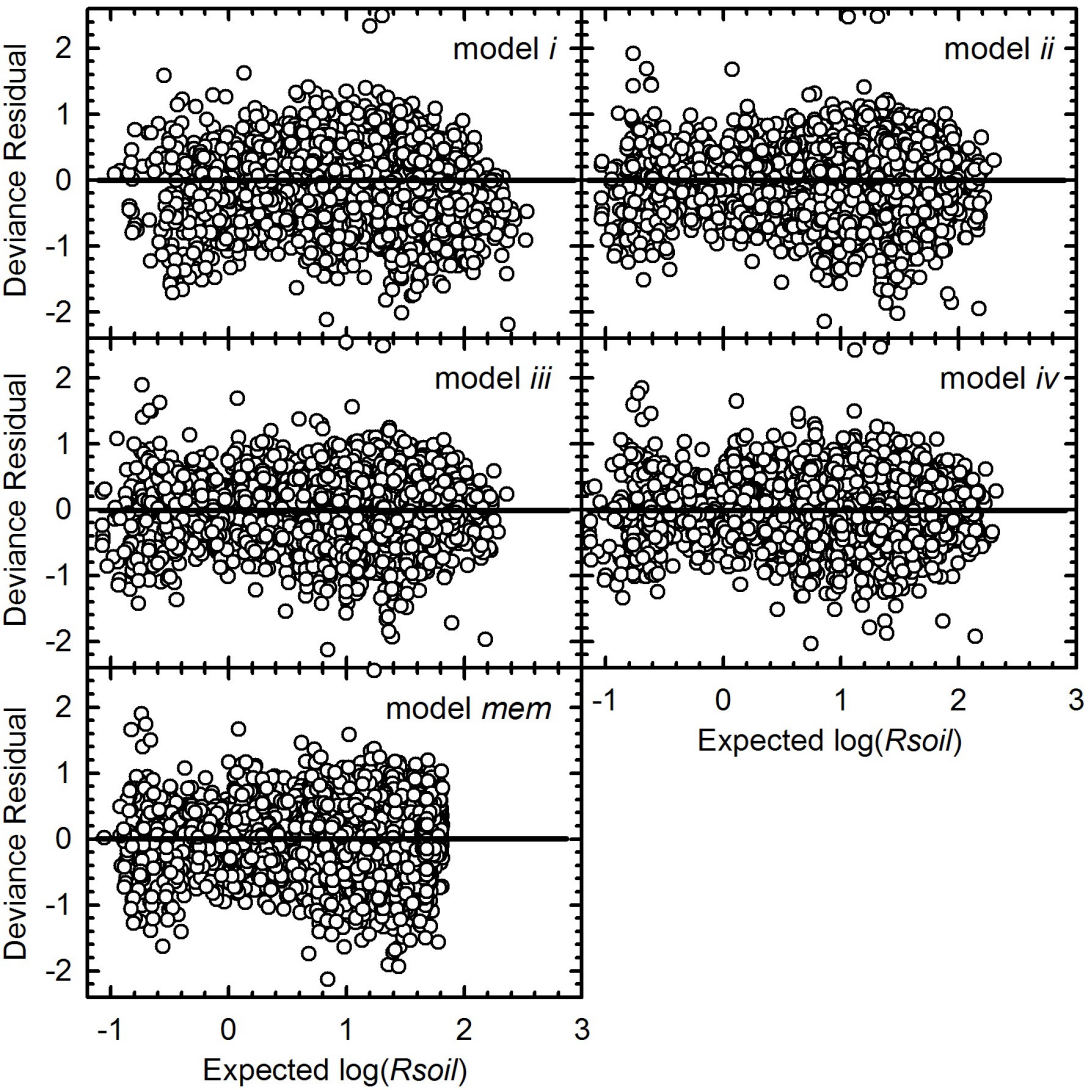
Deviance residuals of the five tested models, based on all observed data. The main-effects model (*mem*) included only the main effects, without interactions: *Tsoil*, *Tsoil*^2^, VWC, VWC^2^, EVI and *Tair*. Horizontal lines divide each plot into positive and negative residuals, for ease of interpretation: the sample size for each is 8039.

Our baseline model *i* confirmed expectations that surface-soil temperature (*Tsoil*, °C) and VWC significantly covaried with *Rsoil* (micromol cm^-2^ s^-1^): they and their interaction were significant at all locations other than La Selva, where their interaction was not informative (S1 Table). Deviance residual plots (Fig 1) did not identify major problems with the model.

Adding either EVI or *Tair* to the baseline model improved model fits at each site: adding them both resulted in model *ii*, which improved model fits at all four sites (Table 3). Both EVI and *Tair* significantly covaried with *Rsoil* at all sites except, again, La Selva, where *in situ* variability in both variables was low. Model *ii* also increased the number of interaction terms by 12. Three of those additional terms were significant at three of the four locations, but none were significant at all four (S1 Table). In addition, in this more complex model, *Tsoil* was rendered non-significant at Bear Creek, and VWC was rendered nonsignificant at Rhodes Farm, although interactions involving each were significant at some locations. Residual plots for fits of model (*ii*) were not obviously better than those of model (*i*).

To test for non-linearities, we individually added quadratic terms for *VWC* and *Tsoil* into model *ii*. Each generated larger model log-likelihoods at each site (Table 3). Model (*iii*) included both quadratic terms, and better fit the observed data at all four sites than did model *ii* (*p* < 0.0.0004, S1 Table). The important interaction terms differed from those of model *ii*. The linear and quadratic terms for both soil temperature and soil moisture were significant at all four locations. Interestingly, the presence of these squared terms shifted the *p* values for the interactions. For example, in comparison to model *ii*, the significance of the *Tsoil* × *Tair* interaction was lost at two locations in model *iii* but emerged at the other two (S1 Table). The deviance residuals from model *iii* were similar to those of model *ii* (Fig 1).

Model *iv* included all terms from model (*iii*) plus the categorical variable land cover, of which there were two at Rhodes Farm and Bear Creek, five at Chequamegon, and four at La Selva. Although the number of parameters was again increased, log likelihoods increased and AIC decreased, at all four locations (Table 3). Land cover was highly significant at all four locations, and interactions between land cover and environmental covariates also occurred at each location (S1 Table). The significance of other interactions was similar in models *iii* and *iv*. Deviance residuals (Fig 1) from model *iv* were similar to those from models *ii* and *iii*.

In proceeding from our baseline model *i* through model *iv*, there was a consistent pattern of lower AIC for more complex models despite increases in the number of parameters (Table 3). That finding was mirrored by likelihood ratio tests of the model sequence, which were entirely nested (S2 Table). Comparisons of model *i* versus model *ii*, model *ii* versus model *iii*, and model *iii* versus model *iv* had *p* values of less than 10^−5^ in 80% of cases and were always significant. The more complex model was favored in each case at each step.

In addition, each of models *i*-*iv* was fit to a data set that combined measurements from all four locations into one collection with 8039 observations (Table 3), which was analyzed without regard to location. The results of these models were used to conduct likelihood ratio tests of full models with location-specific parameters against reduced models with a common set of parameters across all four locations (S2 Table). The maximized log likelihoods for the full models are obtained as a sum across locations of those values. The maximized log likelihood for the reduced model is obtained by fitting the combined data. For each of the five models considered, these tests were highly significant (*p*-values less than 10^−5^), with the result that in each case we would reject the combined model and favor of the location-specific models.

### Main-effects model

With Objective 3 we sought to identify a common model form that included only consistently influential independent variables, even if that was not the model of choice for each location based on AIC and likelihood ratio tests. The main-effects model that emerged (model *mem*, Table 4) contained only the consistently informative covariates *Tsoil*, VWC, EVI, and *Tair*. It did, notably, include quadratic terms for both soil temperature and soil moisture. All terms in this model were significant for three of the four locations, with only VWC and VWC^2^ at Bear Creek as exceptions. Given this consistency, we can examine the estimated parameter values for these covariates (Table 4). There is some consistency in estimated parameter values over locations, although a likelihood ratio test of separate models for individual locations versus a single model for combined locations was significant (*p* value less than 10^−5^, S2 Table), so that we would reject the combined model and conclude that separate parameter values for each location would be preferred. At all sites and in the combined model, all intercepts were < -1.0, at which *Rsoil* equaled 0.37 micromol cm^-2^ s^-1^. Perhaps the most important indication from the parameter values in Table 4 is that all significant squared terms for soil temperature and soil VWC were negative, whereas all other terms were positive, suggesting again that *log*(*Rsoil*) has concave relationships with both *Tsoil* and VWC. This inference was supported by application of the main-effects model (*mem*) to the combined dataset (column 6), in which all included covariates, as well as quadratic terms for soil moisture and temperature, were highly significant.

**Table 4:** Summary of the main-effects model.

	Bear Creek	Chequamegon	La Selva	Rhodes Farm	Combined
Sample size (*N*)	1332	2667	2497	1543	8039
Parameters	7	7	7	7	7
AIC	1892	2443	1889	1309	8271
Log Likelihood	-938	-1213	-937	-646	-4127
model *p*	<0.0001	<0.0001	<0.0001	<0.0001	<0.0001
Parameter *p* values					
Intercept	<0.0001	<0.0001	0.0003	<0.0001	<0.0001
*Tsoil*	<0.0001	<0.0001	0.0002	<0.0001	<0.0001
*Tsoil*^*2*^	<0.0001	<0.0001	0.0002	<0.0001	<0.0001
VWC	0.6315	<0.0001	<0.0001	0.0004	<0.0001
VWC^*2*^	0.1681	<0.0001	<0.0001	<0.0001	<0.0001
EVI	<0.0001	0.0153	0.0049	<0.0001	<0.0001
*Tair*	<0.0001	<0.0001	0.0038	<0.0001	<0.0001
Coefficients					
Intercept	-1.0685	-1.4486	-11.8412	-1.100	-1.1259
*Tsoil*	0.1421	0.1966	0.9832	0.0876	0.1450
*Tsoil*^*2*^	-0.0032	-0.0038	-0.0198	-0.0022	-0.0032
VWC	NS	3.2431	2.4720	3.4658	1.7436
VWC^*2*^	NS	-5.0377	-3.5440	-7.2015	-3.2219
EVI	1.2946	0.2185	0.5601	1.4371	0.8278
*Tair*	0.0240	0.01251	0.0186	0.0297	0.0192

The same model was fit individually to data from four locations, and to the combined dataset. NS indicates not significantly different from zero

## Discussion

Our over-arching goal was to identify environmental correlates of *in situ* soil respiration that meaningfully improved understanding of soil-CO_2_ emission variability within and across four locations, from which multiannual field measurements were made using comparable techniques. Each location was unique in terms of soils, vegetation, or climate (Table 1). We were specifically interested in determining (Objective 1) whether *Rsoil* continued to increase at ever-warmer temperatures, when the joint influences of other environmental covariates were included. By including multiple locations and years in our analyses, we sought to broaden the ranges of environments included, to better characterize which covariates were commonly important, and the forms of their effects. We summarize the models we applied in Table 3, and parameter significance levels for the main models (*i*, *ii*, *iii*, and *iv*) in S1 Table.

### Multiple factors influenced CO_2_ emission rates

Our baseline model *i* proved consistent across locations, with soil temperature and soil water content being significant everywhere, as is widely observed, and their interaction being significant except at one location with uniformly warm and moist soils (La Selva, S1 Table). To address Objective 2, we incorporated the enhanced vegetation index (EVI) and mean daily air temperature (*Tair*) within model *ii*, as potential covariates of canopy processes that contribute to shoot-to-root carbon fluxes, thus broadening the model to potentially include the effects of plant C assimilation. Ambient temperature controls rates of net leaf photosynthesis across plant types [57], independently of leaf area. We found that including *Tair* improved model performance even when *Tsoil* was already included (Table 3) and despite that *Tair* and *Tsoil* correlated strongly (Table 2). The EVI is a widely available, remotely sensed optical measurement of canopy greenness that incorporates influences of total leaf chlorophyll, leaf area, canopy cover, and canopy structure. The use of spectral imagery to improve local-scale *Rsoil* models has previously been demonstrated [58–60]. We cannot conclude cause-and-effect from these results, but inclusion of both EVI and *Tair* improved model performance (S1 Table), possibly by reflecting shoot-to-root carbon fluxes. Our results demonstrate that including both EVI and *Tair* within models can improve local-scale estimations of *Rsoil*, as they do in larger-scale studies [e.g., 61–64].

We did not test the importance of all potentially important covariates of *Rsoil*, but we demonstrated that VWC, *Tsoil*, EVI, and *Tair* were consistently influential. We addressed Objective 2, the potential for non-linearity in the soil temperature-respiration relationship, when the effects of other covariates were included, by adding quadratic terms for both *Tsoil* and VWC to model *ii*, generating model *iii*, with six potential covariates. Again, model performance was improved. Both soil temperature and its quadratic term were significant at all four locations. Both soil moisture and its quadratic term were also significant at all four locations (S1 Table). Throughout these steps the importance of individual covariates and their interactions differed among locations and from model to model, and even their signs, + or -, sometimes switched.

Adding a categorical descriptor of the plot-scale land cover being studied, generating model *iv*, further improved model fits at all four locations (S1 Table). Land cover had the single lowest *p* value at Bear Creek, Chequamegon, and La Selva, and *p* = 0.006 at Rhodes Farm. Model *iv* demonstrated that land cover type significantly influenced rates of soil CO_2_ emission within locations, even after we incorporated multiple location-specific environmental attributes as covariates. We conclude that land cover is very important to include in analyses of emission rates, as reported by others [10, 60, 65, 66].

Our overall goal was to identify the joint influences of different environmental factors on the magnitudes and variability of total *in situ* CO_2_ emissions, which reflect both within-soil CO_2_ production and its release into the atmosphere. The results of our empirical modeling exercise are best interpreted in terms of patterns that emerged, or were absent, over the progression of models considered. It was not our objective to determine “the best” model for relating soil respiration to all the plausible covariates that have been identified in one or another site, but to characterize the effects of previously recognized covariates. We found the *Tsoil*, VWC, their squared terms, EVI, *Tair*, and land cover all improved estimations of *Rsoil*, in comparison to models containing fewer of those covariates (S2 Table).

### Interactions

We included interaction terms in models not to estimate how one effect changed at different levels of another, the traditional interpretation of interactions, but rather as an indication of complexity in the underlying mechanisms that may be involved in determining CO_2_ emission from soils. Throughout, we sought to identify stable and meaningful effects of potential covariates across locations. Our data sets were large enough to support models with multiple covariates. We did not, however, expect measures of model fits, based on likelihood ratio tests and AIC, to continue to favor models containing more terms and more interactions. Model *i* contained four parameters with one interaction term; model *iv* contained an average of 29 parameters including 15 interaction terms; yet at each location and at each step, model log likelihoods increased and AIC decreased as model complexity increased (Table 3).

Throughout the progression of model *i* to model *iv* the number of interaction terms increased, from one to >22. The significance of these interaction terms was apparently haphazard, with most terms being flagged as meaningful at some location, but only two interactions being meaningful at all four study locations: *Tsoil*×EVI in model *ii*; and *Tsoil*×EVI×*Tair* in models *iii* and *iv* (S1 Table). For example, the *Tsoil*×VWC interaction was significant at Chequamegon National Forest in models *i*, *ii* and *iv* but not in model *ii*, where *p* > 0.2. Even the nature of its effect, positive or negative, varied (S1 Table). The *Tsoil*×VWC interaction was never significant at La Selva but was influential in all four models at Bear Creek. The indication is that land cover and interaction terms that were added to basic models were picking up some structure present in location-specific data sets, but that structure was not necessarily related in any broadly applicable mechanistic fashion to the physical covariates involved. Overall, the lack of a pattern in the significance of interaction terms suggests that there were complex relations among the primary variables of *Rsoil*, *Tsoil*, VWC, *Tair*, and EVI at individual study locations, and that those relationships were not necessarily consistent across land-cover types or geographic locations.

### Marginal effects and maximal responses

The conclusion of the preceding paragraph suggested that it might be profitable to examine marginal models which, although they may not provide the “best fit” at any individual location, retain some stability in meaningful terms and estimated parameters (Objective 3). Our analysis indicated that, for the four locations considered in this work, that model included soil temperature and soil water content, along with their squared terms, and EVI and air temperature:

Main-effects model
$$\hat{\lambda }=-1.1259+0.1450\;Tsoil–0.0032\;{Tsoil}^{2}+1.7436\;VWC–3.2219\;{VWC}^{2\;}+0.8278\;EVI+0.0192\;Tair$$

In this model, the parameter values were visually stable in sign and rough magnitudes over locations, although formal tests indicate we would still prefer to estimate parameters for this model separately for each location (S1 Table). Of particular interest for this marginal model, both soil temperature and soil water content appear to have marginal concave relations with soil respiration.

**Fig 2:**
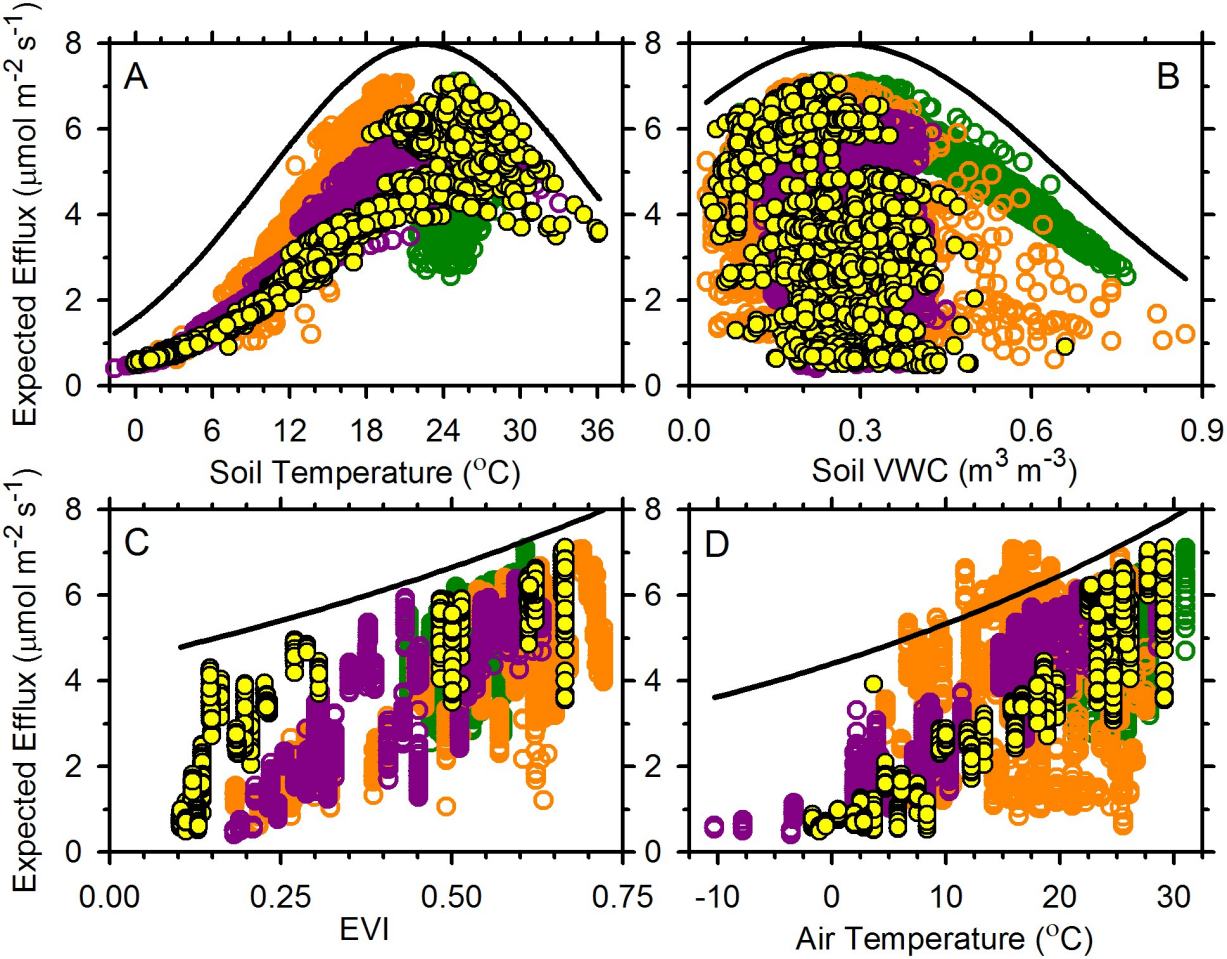
Expected soil-CO_2_ emissions across observed ranges of (A) surface-soil temperature; (B) surface-soil volumetric water content; (C) enhanced vegetation index (EVI); and (D) air temperature. Expected values are based on the main effects model applied to the observed environments (*N* = 8039). Symbol colors represent locations: Bear Creek—orange; Chequamegon—purple; La Selva—green; and Rhodes Farm—yellow.

To visualize these marginal relations, we plotted the expected values of *Rsoil* (i.e., the regression predicated values) from the marginal regression fit to the combined data (Table 4) against the observed values of the individual covariates of *Tsoil*, VWC, EVI, and *Tair* (Fig 2). These plots indicate a type of limiting effect of each covariate on the expected response, as by [67]. Maximal expected responses (for observed values of the other covariates) appear to be related in a monotonic fashion for both EVI and *Tair*, but in an intermediate-optimum fashion for *Tsoil* and VWC. The exact estimated expectations are functions of all four covariates as a set, and most of the expected values fail to obtain the largest values possible, being scattered below the maximal or limiting curve. The estimated expectations (mu; hat) plotted on the vertical axes of the graphs in Fig 2 were computed as:

$$mu;hat=exp\left[lambda;hat+½{sigma;hat}^{2}\right]$$ (2)

where lambda;hat represents the regression-based estimate of log(*Rsoil*), and sigma;hat^2^ represents its variance (*N* = 8039). The value of EVI at which an overall maximum occurs will simply be the largest value of EVI observed (i.e., 0.7214) and the same is true for *Tair* (31.01°C). We can determine the values of *Tsoil* and VWC at which overall maximums will occur by setting partial derivatives equal to zero, which gives 0.27 m^3^ m^-3^ for VWC and 22.5°C for *Tsoil*. These match well visually with Fig 2.

In this main-effects model, both EVI and air temperature covaried positively with *Rsoil* (Fig 2C and 2D), to maxima defined by the upper ranges of the observed data. Given that caveat we are unable to guess what might happen if either was greater. We gathered data from multiple sites to enhance the breadth of environments included, but the use of observed data to forecast possible futures faces this problem, i.e., the inability to address unobserved conditions. However, including EVI and *Tair* into our models improved model fits to the observed data (S2 Table), indicating that they were meaningful covariates with *Rsoil*. Altogether we found that *Rsoil* was potentially limited by four environmental factors (Fig 2), and was affected by the land cover (Table 3). These findings are consistent with our understanding of *Rsoil* as a system-level flux that derives from multiple interacting organisms and processes, each of which may be influenced distinctly by environmental conditions.

Our analysis also indicated that soil-CO_2_ emissions varied quadratically with soil moisture. That is consistent with previous observations that maximum CO_2_ production occurs at intermediate soil moisture contents [47, 68–75]. Soil water content influences vegetation productivity, detritus decay rates, and soil-atmosphere gas exchanges. Dry conditions diminish the availability of dissolved substrates to soil organisms, and directly depress cellular expansion and replication [69, 70]. In wet soils, water-filled pores diminish soil-atmosphere gas exchange, whereas a lack of air-filled pore volume may limit oxygen availability to soil organisms and thereby inhibit aerobic respiration [71, 76–78]. In our study, relatively low soil water contents were observed at all three temperate locations, but they did not depress CO_2_ emissions to the same extent as high soil water contents did. The optimal soil water content for CO_2_ emission across the sites was 0.27 m^3^ m^-3^, with emission rates declining in both drier and wetter soils (Fig 2B). That compares well with the ~27% global optimum inferred by [79], but we expect that the relationship will vary among soil types, textures, and organic matter contents [80].

We specifically sought, with Objective 1, to determine if *Rsoil* increased monotonically with increasing soil temperature, when additional covariates were considered. We found that emissions did strongly increase as soils warmed, but only to a maximum at ca. 22.5°C across locations. As soils warmed beyond that, emission rates neither increased further nor plateaued—they declined (Fig 2A). Slightly warmer temperature optima were inferred by [6], i.e., ~25°C across nine biomes; and by [81], i.e., 24.1–27.4°C for subtropical ecosystems. In our case, expected emissions at 30°C were the same as those at 15°C. Why might soil respiration decline as soils warm? We did not generate any evidence that soil heterotrophic respiration declined as soils warmed, whereas many studies have demonstrated that temperature optima for soil microbial respiration are higher than the temperatures we observed [82–86]. Our finding that *Rsoil* declined beyond an optimum temperature was robust in all four locations in our study (S1 Table, models *iii*–*iv*).

**Fig 3:**
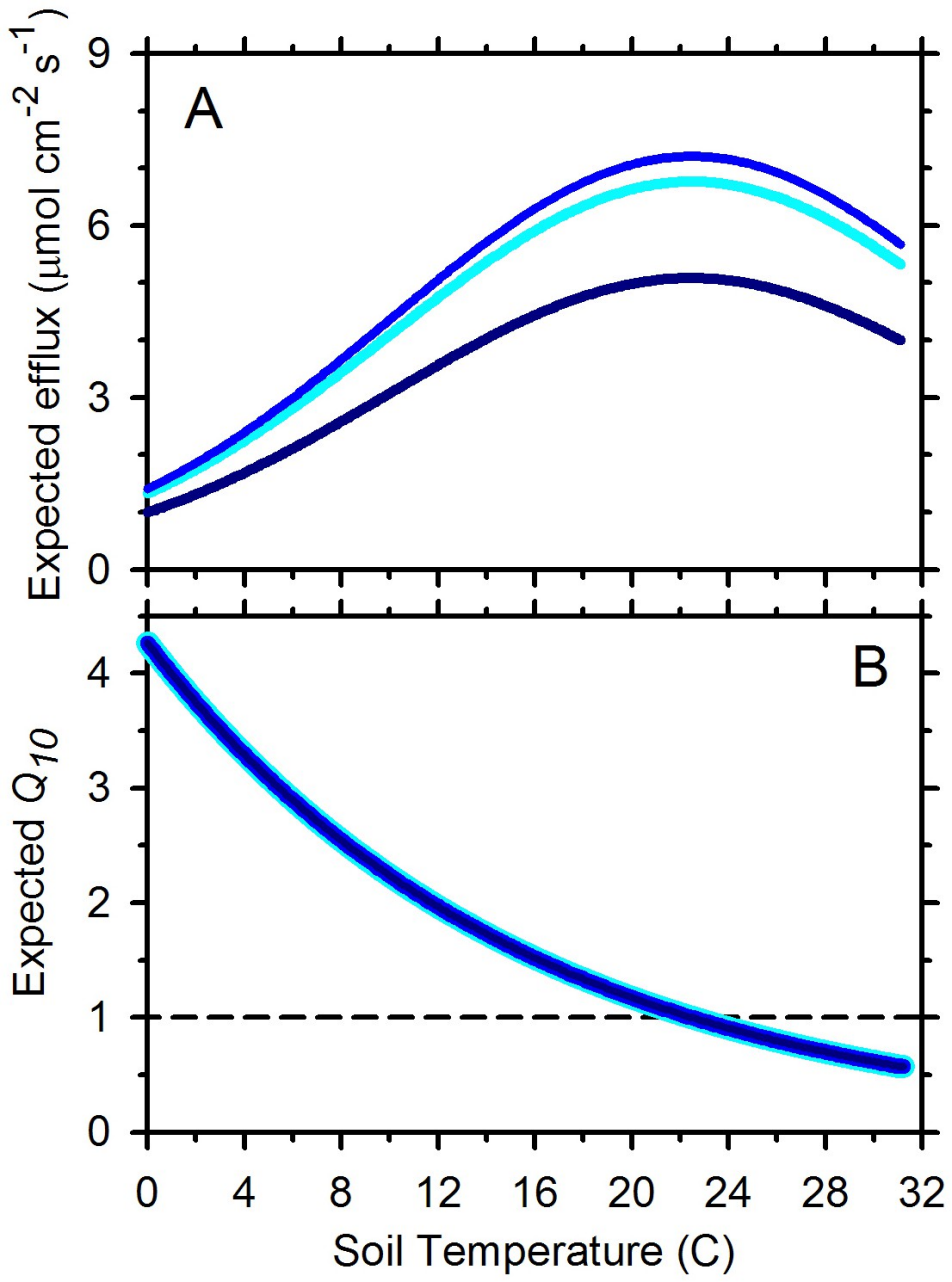
Expected relationships between soil-CO_2_ emissions and temperature at three levels of soil volumetric water content based on the main-effects model. (A) Expected *Rsoil* in relation to soil temperature at low, medium and high soil volumetric water contents. The temperature at maximum flux was 22.5°C regardless of soil water content. (B) The *Q*_*10*_ of emissions declined as temperature increased, but did not vary with water content. The plotted relationships show the midpoints of 10°C intervals, *i*.*e*., the *Q*_*10*_ at X = 2°C is the ratio of the expected fluxes at 7 and -3°C. Note that the individual relationships for low, medium, and high water contents overlie one another. The horizontal dotted line identifies *Q*_*10*_ = 1, below which estimated fluxes were lower at warmer temperatures.

To pursue this question further, we evaluated the form of the relationship between temperature and the *Q*_*10*_ of *Rsoil*, by applying the main-effects model to generate expected values of *Rsoil* at soil volumetric water contents (m^3^ m^-3^) of 0.13 (10^th^ percentile), 0.27 (optimum), and 0.52 (90^th^ percentile), across a soil temperature range of 0°C to 31°C, given maximum observed values of EVI and *Tair*. We found that the Q_10_ of *Rsoil* was not affected by soil water content, consistent with [87], although its magnitude was (Fig 3). Reported *Q*_10_ values for *Rsoil* vary among studies [88], but usually within the range of 1–5 [66, 89–91]. In our case, the expected *Q*_*10*_ of emissions varied from 0.57 to 4.27: it declined from an average of 4.3 at 0°C, to 2.0 at 12°C, to 0.6 at 30°C (Fig 3B). The expected across-site temperature optimum for *Rsoil* occurred at 22.5°C, where the expected *Q*_*10*_ equaled 1.0; emission rates thereafter declined at warmer temperatures. Similar results are presented elsewhere [62, 89, 92]. Based on this analysis, the *Q*_*10*_ of *Rsoil* varied broadly, even within sites, and its magnitude varied with temperature but not moisture. This finding results from the significantly negative *Tsoil*^2^ terms in our models, but potentially explains, in part, variability in observed *Rsoil Q*_*10*_ values within and among studies.

Our results seem to highlight the importance of temperate-zone seasonality: air temperatures <30.1°C, the maximum observed temperature within our dataset, were associated with lower soil-CO_2_ emissions (Fig 2D). The effects of EVI (Fig 2C) were similar: both EVI and air temperature were low at the beginning of the growing seasons in the temperate sites and increased as growing seasons progressed. Unfortunately, our dataset included only 38 observations at air temperature of ≥ 30°C, and those were all at one location on one day. The upper limit for positive net CO_2_ uptake by woody and many temperate-zone plants typically ranges from ca. 35–50°C [57, 81], beyond the range of our observations. We therefore cannot address the effects of air temperatures higher than 30°C on *Rsoil* or its autotrophic component. Pertinently, the air-temperature optima of vegetation productivity (i.e., estimated gross primary productivity) were found to be consistently lower than those for leaf photosynthesis, with a peak at 23°C [63], or with peaks at 18 and 28°C for C_3_ and C_4_ vegetation [93]. Those findings are similar to our across-location estimate of 22.5°C for *Rsoil*, suggesting—or confirming—that plant productivity influences soil CO_2_ emissions.

### Models and meaning

Our analyses were consistent in demonstrating that location-specific analyses provided better model fits to observed data than did models based on the same data but excluding location (Table 3, S1 and S2 Tables). Our results suggest that incorporating local-scale information about EVI and *Tair*, in addition to *Tsoil* and VWC, can improve local-scale analyses of *Rsoil*: quantitative data for both variables are widely available. Many other environmental variables have been shown to covary significantly with in situ *Rsoil* and might be useful at other locations: further studies of additional variables and other locations are needed. Our results do demonstrate that incorporation of *Tsoil*, *Tair*, VWC, and EVI can be useful for empirical modeling of in situ soil-CO_2_ emissions at local and broader scales, and that quadratic terms for *Tsoil* and VWC can improve model fits. We also found that land cover typically had the single-most highly significant influence on *Rsoil*, within sites, even after multiple bioclimatic variables were included (Table 3, S1 Table). That indicates that models should distinguish among land cover types and experimental treatments whenever possible, to improve analyses. Given these findings, we sought insights into broader-scale questions. We found that a main-effects model that included only consistently meaningful covariates, without interactions, consistently provided interpretable findings across sites, despite that it was never ‘the best’ model at any one site.

Soil-CO_2_ emission constitutes a major land-to-atmosphere carbon flux that is highly variable over land use and vegetation types and is responsive to climatic conditions. That soil-CO_2_ emissions can be measured directly, in contrast to processes such as gross and net primary productivity and ecosystem respiration, allows the development of empirically derived models for land-to-atmosphere flux that can be used to test model predictions, and perhaps suggest modifications to those models [94, 95]. In the development of purely empirical models of soil-CO_2_ emissions it is important to distinguish between models that seek to provide the best possible fit to a data set of limited spatial and temporal scope, and models that seek to provide insight into the most consistently important factors across the range of situations in which data are collected. These consistently important factors, if they exist, become candidates for ubiquitous mechanistic forces that drive the system. We show that soil-CO_2_ emissions at local levels are influenced by a variety of potentially interacting variables, and by many possible interactions that exist among those variables. We show that a much smaller number of covariates in a model containing only main effects is stable across situations, meaning that the significance of the variables and their estimated coefficients are consistent among regression models estimated from different data sets. We also demonstrate that soil moisture and temperature relationships are significantly non-linear: emissions may be expected to either increase or decline if temperatures and rainfall increase, depending upon their prior conditions. Recognizing that local soil-CO_2_ emissions are influenced by multiple situation-specific limiting factors, but that a smaller and consistently important set of widely limiting factors may exist, can benefit terrestrial carbon-cycling analyses across scales.

## Supporting information

S1 Table Significance of model parameter values at four locations. The right column refers to the same model applied to the data from all four sites without reference to location. An NS indicates not significantly different from zero.
(DOCX)

S2 Table Likelihood Ratio tests of differences between models, where Model 1 is nested within Model 2. The chi-squared and *p*-values refer to one-sided tests of the null hypothesis that the log-likelihood of Model 2 is not different from that of Model 1.
(DOCX)
