# Supplementary material for: Multiple factors co-limit short-term in situ soil carbon dioxide emissions
Source: PLoS One. 2023 Feb 15;18(2):e0279839. doi: 10.1371/journal.pone.0279839 (PMC9931153; doi:10.1371/journal.pone.0279839)
Supplement: S1 Table — The right column refers to the same model applied to the data from all four sites without reference to location. An NS indicates not significantly different from zero. (DOCX) [file pone.0279839.s001.docx]

**S1 Table.** **Significance of model parameter values at four locations.**  The Combined column, at right, was derived from the data from all four locations without reference to location. An NS indicates that those parameter values are not significantly different from zero (*p* < 0.05).

|  | Bear Creek | Chequamegon | La Selva | Rhodes Farm | Combined |
| --- | --- | --- | --- | --- | --- |
| Samples (*N*) | 1332 | 2667 | 2497 | 1543 | 8039 |
| Model *i*, variables *Tsoil*, VWC | | | | | |
| No. of Parameters | 4 | 4 | 4 | 4 | 4 |
| AIC | 2160 | 2788 | 2021 | 1802 | 10927 |
| Log Likelihood | -1075 | -1389 | -1006 | -896 | -5459 |
| Parameter *p*: |  |  |  |  |  |
| Intercept | <0.0001 | <0.0001 | <0.0001 | 0.43 | <0.0001 |
| *Tsoil* | <0.0001 | <0.0001 | 0.0002 | <0.0001 | <0.0001 |
| VWC | 0.0030 | <0.0001 | <0.0001 | 0.0026 | <0.0001 |
| *Tsoil*×VWC | <0.0001 | 0.0003 | 0.74 | <0.0001 | <0.0001 |
| Parameter values: |  |  |  |  |  |
| Intercept | -0.6358 | -0.5500 | 1.0417 | NS | 0.2036 |
| *Tsoil* | 0.0959 | 0.1165 | 0.0283 | 0.0789 | 0.0725 |
| VWC | 0.5827 | 0.4460 | -0.6682 | -0.4642 | -0.9426 |
| *Tsoil*×VWC | 0.1612 | 0.0623 | NS | 0.1745 | -0.0340 |
| Model *ii*, variables *Tsoil*, VWC, EVI, *Tair* | | | | | |
| No. of Parameters | 16 | 16 | 16 | 16 | 16 |
| AIC | 1831 | 2671 | 1937 | 1263 | 8637 |
| Log Likelihood | -898 | -1318 | -951 | -614 | -4302 |
| Parameter *p*: |  |  |  |  |  |
| Intercept | 0.0066 | <0.0001 | 0.0009 | 0.76 | <0.0001 |
| *Tsoil* | 0.23 | <0.0001 | 0.0007 | 0.0089 | <0.0001 |
| VWC | 0.0082 | 0.0250 | <0.0001 | 0.24 | <0.0001 |
| EVI | 0.0001 | 0.0001 | 0.11 | <0.0001 | <0.0001 |
| *Tair* | 0.0039 | 0.0032 | 0.30 | <0.0001 | <0.0001 |
| *Tsoil*×VWC | <0.0001 | 0.20 | 0.0401 | 0.0003 | <0.0001 |
| *Tsoil*×EVI | <0.0001 | <0.0001 | 0.0027 | 0.0032 | <0.0001 |
| *Tsoil*×*Tair* | 0.64 | 0.20 | 0.0016 | <0.0001 | <0.0001 |
| VWC×EVI | <0.0001 | 0.0512 | <0.0001 | 0.83 | 0.0380 |
| VWC×*Tair* | 0.08 | 0.16 | 0.0117 | 0.61 | 0.30 |
| EVI×*Tair* | 0.08 | <0.0001 | <0.0001 | 0.0035 | <0.0001 |
| *Tsoil*×VWC×EVI | 0.65 | 0.31 | 0.0277 | 0.09 | <0.0001 |
| *Tsoil*×VWC×*Tair* | 0.17 | 0.72 | 0.88 | 0.37 | 0.17 |
| *Tsoil*×EVI×*Tair* | 0.0005 | 0.0008 | 0.0058 | 0.20 | 0.0084 |
| VWC×EVI×*Tair* | 0.0007 | 0.58 | 0.70 | 0.0273 | 0.0015 |
| *Tsoil*×VWC×EVI×*Tair* | 0.38 | 0.31 | 0.48 | <0.0001 | 0.0475 |
| Parameter values: |  |  |  |  |  |
| Intercept | 0.6611 | -0.6297 | 0.8175 | NS | 0.2907 |
| *Tsoil* | NS | 0.0979 | 0.0397 | 0.0111 | 0.0265 |
| VWC | -1.6053 | 0.2636 | -0.6741 | NS | -0.2630 |
| EVI | 1.4221 | 0.4113 | NS | 1.4649 | 0.7218 |
| *Tair* | 0.0224 | 0.0106 | NS | 0.0300 | 0.0170 |
| *Tsoil*×VWC | 0.3662 | NS | -0.1915 | 0.2012 | -0.0844 |
| *Tsoil*×EVI | -0.2334 | -0.2285 | 0.9027 | 0.0835 | -0.0615 |
| *Tsoil*×*Tair* | NS | NS | -0.0176 | -0.0015 | -0.0026 |
| VWC×EVI | -23.8329 | NS | 11.6390 | NS | -0.9521 |
| VWC×*Tair* | NS | NS | 0.1733 | NS | NS |
| EVI×*Tair* | NS | 0.1156 | 0.7202 | -0.0788 | 0.0656 |
| *Tsoil*×VWC×EVI | NS | NS | 4.5211 | NS | -0.5559 |
| *Tsoil*×VWC×*Tair* | NS | NS | NS | NS | NS |
| *Tsoil*×EVI×*Tair* | 0.0144 | -0.0112 | 0.4217 | NS | 0.0018 |
| VWC×EVI×*Tair* | 1.8723 | NS | NS | -0.8020 | 0.3203 |
| *Tsoil*×VWC×EVI×*Tair* | NS | NS | NS | -0.1040 | 0.0121 |
| Model *iii*, variables: *Tsoil*, *Tsoil^2^*, VWC, VWC*^2^*, EVI, *Tair* | | | | | |
| No. of Parameters | 18 | 18 | 18 | 18 | 18 |
| AIC | 1817 | 2400 | 1857 | 1251 | 8170 |
| Log Likelihood | -889 | -1181 | -909 | -606 | -4066 |
| Parameter *p*: |  |  |  |  |  |
| intercept | <0.0001 | <0.0001 | 0.0005 | 0.0003 | <0.0001 |
| *Tsoil* | 0.0033 | <0.0001 | 0.0004 | 0.0031 | <0.0001 |
| *Tsoil^2^* | 0.0006 | <0.0001 | 0.0005 | 0.0098 | <0.0001 |
| VWC | 0.0002 | <0.0001 | <0.0001 | 0.0071 | <0.0001 |
| VWC*^2^* | 0.0218 | <0.0001 | <0.0001 | 0.0027 | <0.0001 |
| EVI | 0.0007 | 0.0001 | 0.09 | <0.0001 | <0.0001 |
| *Tair* | <0.0001 | 0.0004 | 0.72 | <0.0001 | <0.0001 |
| *Tsoil*×VWC | <0.0001 | <0.0001 | 0.25 | 0.0488 | <0.0001 |
| *Tsoil*×EVI | <0.0001 | 0.84 | 0.0002 | 0.0002 | 0.0339 |
| *Tsoil*×*Tair* | 0.0177 | 0.0016 | 0.35 | 0.78 | 0.52 |
| VWC×EVI | <0.0001 | 0.0014 | 0.0005 | 0.56 | <0.0001 |
| VWC×*Tair* | 0.0366 | 0.68 | 0.59 | 0.77 | 0.0019 |
| EVI×*Tair* | 0.0005 | 0.36 | 0.15 | 0.0006 | 0.62 |
| *Tsoil*×VWC×EVI | 0.65 | 0.58 | 0.09 | 0.0376 | <0.0001 |
| *Tsoil*×VWC×*Tair* | 0.0505 | 0.24 | 0.47 | 0.39 | 0.28 |
| *Tsoil*×EVI×*Tair* | 0.0003 | 0.0124 | 0.0071 | 0.0426 | 0.0429 |
| VWC×EVI×*Tair* | <0.0001 | 0.15 | 0.87 | 0.0301 | 0.96 |
| *Tsoil*×VWC×EVI×*Tair* | 0.23 | 0.06 | 0.14 | 0.0001 | 0.0459 |
| Parameter values: |  |  |  |  |  |
| intercept | 1.7507 | -2.4177 | -20.7809 | -0.8731 | -1.2427 |
| *Tsoil* | -0.0895 | 0.3157 | 1.7161 | 0.0770 | 0.1531 |
| *Tsoil^2^* | 0.0028 | -0.0079 | -0.0340 | -0.0020 | -0.0036 |
| VWC | -4.0526 | 3.1889 | 2.3364 | 2.7664 | 1.8746 |
| VWC*^2^* | 3.8659 | -5.4455 | -3.3853 | -5.6874 | -3.0939 |
| EVI | 1.2551 | 0.3919 | NS | 1.3913 | 0.8491 |
| *Tair* | 0.0326 | 0.0120 | NS | 0.0280 | 0.0212 |
| *Tsoil*×VWC | 0.4577 | -0.1914 | NS | 0.1173 | -0.0584 |
| *Tsoil*×EVI | -0.3525 | NS | 1.1272 | 0.1696 | 0.0252 |
| *Tsoil*×*Tair* | -0.0031 | 0.0033 | NS | NS | NS |
| VWC×EVI | -27.5637 | 3.4668 | 6.7287 | NS | -1.7955 |
| VWC×*Tair* | 0.1834 | NS | NS | NS | 0.0477 |
| EVI×*Tair* | 0.2180 | NS | NS | -0.1191 | NS |
| *Tsoil*×VWC×EVI | NS | NS | NS | 0.7899 | -0.4721 |
| *Tsoil*×VWC×*Tair* | NS | NS | NS | NS | NS |
| *Tsoil*×EVI×*Tair* | 0.0149 | -0.0079 | 0.4055 | 0.0031 | 0.0013 |
| VWC×EVI×*Tair* | 2.4764 | NS | NS | -0.8029 | NS |
| *Tsoil*×VWC×EVI×*Tair* | NS | NS | NS | -0.0890 | -0.0121 |
| Model (*iv*), variables *Tsoil*, *Tsoil^2^*, VWC, VWC*^2^*, EVI, *Tair,* CoverType | | | | | |
| No. of Parameters | 23 | 38 | 33 | 23 | 68 |
| AIC | 1624 | 2122 | 1534 | 1246 | 7148 |
| Log Likelihood | -787 | -1021 | -732 | -599 | -3505 |
| Parameter *p*: |  |  |  |  |  |
| intercept | <0.0001 | 0.65 | 0.0484 | 0.0002 | 0.83 |
| *Tsoil* | 0.0045 | <0.0001 | 0.0434 | 0.0012 | 0.0006 |
| *Tsoil^2^* | <0.0001 | <0.0001 | 0.0436 | 0.0046 | 0.12 |
| VWC | <0.0001 | <0.0001 | <0.0001 | 0.0174 | <0.0001 |
| VWC*^2^* | <0.0001 | <0.0001 | <0.0001 | 0.0087 | <0.0001 |
| EVI | 0.0305 | 0.60 | 0.0086 | <0.0001 | 0.17 |
| *Tair* | 0.0003 | 0.70 | 0.0487 | <0.0001 | 0.92 |
| *Tsoil*×VWC | <0.0001 | <0.0001 | 0.20 | 0.08 | 0.57 |
| *Tsoil*×EVI | <0.0001 | 0.12 | 0.0033 | <0.0001 | <0.0001 |
| *Tsoil*×*Tair* | 0.0011 | 0.17 | 0.86 | 0.93 | 0.0128 |
| VWC×EVI | <0.0001 | 0.10 | 0.0017 | 0.42 | <0.0001 |
| VWC×*Tair* | 0.23 | 0.09 | 0.92 | 0.88 | 0.19 |
| EVI×*Tair* | <0.0001 | 0.48 | 0.40 | 0.0002 | <0.0001 |
| *Tsoil*×VWC×EVI | 0.42 | 0.32 | 0.07 | 0.0485 | 0.0106 |
| *Tsoil*×VWC×*Tair* | 0.0111 | 0.50 | 0.42 | 0.49 | 0.7241 |
| *Tsoil*×EVI×*Tair* | 0.0028 | 0.0208 | 0.0404 | 0.0397 | <0.0001 |
| VWC×EVI×*Tair* | <0.0001 | 0.55 | 0.87 | 0.0202 | 0.73 |
| *Tsoil*×VWC×EVI×*Tair* | 0.14 | 0.11 | 0.0430 | 0.0002 | 0.13 |
| LandCover * | <0.0001 | <0.0001 | <0.0001 | 0.0060 | <0.0001 |
| LandCover×*Tsoil ** | 0.78 | 0.95 | 0.0002 | 0.06 | <0.0001 |
| LandCover×VWC *** | 0.17 | <0.0001 | <0.0001 | 0.30 | <0.0001 |
| LandCover×EVI * | <0.0001 | 0.09 | 0.31 | 0.18 | <0.0001 |
| LandCover×*Tair ** | 0.10 | 0.32 | 0.0104 | 0.52 | <0.0001 |
| Parameter values: |  |  |  |  |  |
| intercept | 1.9976 | NS | -11.3419 | -0.9274 | NS |
| *Tsoil* | -0.0818 | 0.2633 | 0.9344 | 0.0869 | 0.0589 |
| *Tsoil ^2^* | 0.0030 | -0.0062 | -0.0188 | -0.0023 | NS |
| VWC | -5.6793 | 1.8007 | 3.1676 | 2.4734 | 2.3016 |
| VWC*^2^* | 8.0270 | -3.7951 | -4.3672 | -5.0291 | -3.8443 |
| EVI | 0.7511 | NS | 0.5509 | 1.3778 | NS |
| *Tair* | 0.0301 | NS | 0.0160 | 0.0276 | NS |
| *Tsoil*×VWC | 0.4561 | -0.1570 | NS | NS | NS |
| *Tsoil*×EVI | -0.3408 | NS | 0.8657 | 0.1804 | -0.0916 |
| *Tsoil*×*Tair* | -0.0041 | NS | NS | NS | -0.0009 |
| VWC×EVI | -20.5355 | NS | 5.8295 | NS | -2.3358 |
| VWC×*Tair* | NS | NS | NS | NS | NS |
| EVI×*Tair* | 0.2521 | NS | NS | -0.1287 | 0.0766 |
| *Tsoil*×VWC×EVI | NS | NS | NS | 0.7495 | -0.2704 |
| *Tsoil*×VWC×*Tair* | -0.0233 | NS | NS | NS | NS |
| *Tsoil*×EVI×*Tair* | 0.0114 | -0.0073 | 0.2926 | 0.0031 | 0.0036 |
| VWC×EVI×*Tair* | 2.1088 | NS | NS | -0.8590 | NS |
| *Tsoil*×VWC×EVI×*Tair* | NS | NS | -1.7282 | -0.0864 | NS |

* Based on Effect Tests; parameter values are vegetation specific, and so are not shown.
