# Supplementary material for: Multiple factors co-limit short-term in situ soil carbon dioxide emissions
Source: PLoS One. 2023 Feb 15;18(2):e0279839. doi: 10.1371/journal.pone.0279839 (PMC9931153; doi:10.1371/journal.pone.0279839)
Supplement: S2 Table — The chi-squared and p-values refer to one-sided tests of the null hypothesis that the log-likelihood of Model 2 is not different from that of Model 1. (DOCX) [file pone.0279839.s002.docx]

**S2 Table**. Likelihood ratio tests of differences between models, where Model 1 is nested within Model 2. The *p* values refer to the probability that the log-likelihoods of the two models are not significantly different.

| **Model 1** | **Model 2** | **Location** | ***df*_2_-*df*_1_** | **LR* test statistic** | ***p*** |
| --- | --- | --- | --- | --- | --- |
| Comparisons of increasingly complex models | | | | | |
| model *i* | model *ii* | Bear Creek | 12 | 354 | <10^-5^ |
|  |  | Chequamegon | 12 | 142 | <10^-5^ |
|  |  | La Selva | 12 | 109 | <10^-5^ |
|  |  | Rhodes Farm | 12 | 563 | <10^-5^ |
| model *ii* | model *iii* | Bear Creek | 2 | 18 | <0.0002 |
|  |  | Chequamegon | 2 | 275 | <10^-5^ |
|  |  | La Selva | 2 | 83 | <10^-5^ |
|  |  | Rhodes Farm | 2 | 16 | <0.0005 |
| model *iii* | model *iv* | Bear Creek | 5 | 203 | <10^-5^ |
|  |  | Chequamegon | 20 | 319 | <10^-5^ |
|  |  | La Selva | 15 | 354 | <10^-5^ |
|  |  | Rhodes Farm | 5 | 16 | <0.008 |
| model *mem* | model *iii* | Bear Creek | 16 | 97 | <10^-5^ |
|  |  | Chequamegon | 31 | 65 | <0.0004 |
|  |  | La Selva | 26 | 408 | <0.001 |
|  |  | Rhodes Farm | 16 | 95 | <10^-5^ |
| model *iC* | model *i* | all four | 9 | 2185 | <10^-5^ |
| model *iiC* | model *ii* | all four | 45 | 1039 | <10^-5^ |
| model *iiiC* | model *iii* | all four | 51 | 960 | <10^-5^ |
| model *ivC* | model *iv* | all four | 46 | 729 | <10^-5^ |
| *mem*** | *mem*C | all four | 18 | 786 | <10^-5^ |

* Likelihood Ratio

** main-effects model; *mem*C is the combined (i.e.,

location-free) main-effects model
